# MCtandem: an efficient tool for large-scale peptide identification on many integrated core (MIC) architecture

**DOI:** 10.1186/s12859-019-2980-5

**Published:** 2019-07-17

**Authors:** Chuang Li, Kenli Li, Keqin Li, Feng Lin

**Affiliations:** 1 College of Computer Science and Electronic Engineering, Hunan University, Lushannan Road, Changsha, 410082 China; 2 National Supercomputing Center in Changsha, Lushannan Road, Changsha, 410082 China; 3 Department of Computer Science, State University of New York, New Paltz, New York, 12561 USA; 4 School of Computer Science and Engineering, Nanyang Technological University, Nangyang Road, Singapore, 639798 Singapore

**Keywords:** Peptide identification, Tandem mass spectrometry (MS/MS), Database searching, High performance computing, Many Integrated Core (MIC)

## Abstract

**Background:**

Tandem mass spectrometry (MS/MS)-based database searching is a widely acknowledged and widely used method for peptide identification in shotgun proteomics. However, due to the rapid growth of spectra data produced by advanced mass spectrometry and the greatly increased number of modified and digested peptides identified in recent years, the current methods for peptide database searching cannot rapidly and thoroughly process large MS/MS spectra datasets. A breakthrough in efficient database search algorithms is crucial for peptide identification in computational proteomics.

**Results:**

This paper presents MCtandem, an efficient tool for large-scale peptide identification on Intel Many Integrated Core (MIC) architecture. To support big data processing capability, a novel parallel match scoring algorithm, named MIC-SDP (spectrum dot product), and its two-level parallelization are presented in MCtandem’s design. In addition, a series of optimization strategies on both the host CPU side and the MIC side, which includes pre-fetching, optimized communication overlapping scheme, multithreading and hyper-threading, are exploited to improve the execution performance.

**Conclusions:**

For fair comparisons, we first set up experiments and verified the 28 fold times speedup on a single MIC against the original CPU-based implementation. We then execute the MCtandem for a very large dataset on an MIC cluster (a component of the Tianhe-2 supercomputer) and achieved much higher scalability than in a benchmark MapReduce-based programs, MR-Tandem. MCtandem is an open-source software tool implemented in C++. The source code and the parameter settings are available at https://github.com/LogicZY/MCtandem.

## Background

In the proteomics era, mass spectrometry has become a leading technology for proteomic analysis, including the high-throughput analysis of proteins and determination of their primary structures. Database search-based peptide identification, which aims to retrieve all candidate sequences from a specified protein sequence database for each tandem mass spectrometry (MS/MS) spectrum, is widely used for protein analysis. It can process the peptide sequence and post-translational modifications (PTMs) with high accuracy, sensitivity, and throughput. X!Tandem [1], SEQUEST [2], Mascot [3], pFind [4, 5] and OMSSA [6] are examples of excellent peptide identification tools in proteomics.

However, existing peptide database search tools still suffer from low computational efficiency due to a number of limitations. First, modern mass spectrometers can generate millions of MS/MS spectra in each experiment, which makes matching of these fragmentation spectra to peptides a bottleneck in proteomics research [7] (e.g., entire human proteome identification). Second, the database search criteria have become increasingly demanding, e.g., in semi-unconstrained enzyme searches and/or when considering multiple variable PTMs [8]. Finally, the integration of acquired sequence data into central databases such as Liverwiki [9] typically requires the updating and depositing of a large amount of spectra data files.

Without the development of more powerful and efficient peptide database searching methods, we can expect computational bottlenecks to limit the scope of discoveries to small-scale MS/MS spectra data. Therefore, a breakthrough in efficient database search algorithms is crucial for large-scale peptide identification, especially entire human proteome analysis, in computational proteomics.

Fortunately, various high performance computing (HPC) frameworks and hardware techniques, such as Message Passing Interface (MPI) [10], MapReduce [11], field programmable gate arrays (FPGAs) [12], Intel Many Integrated Core Architecture (MIC) [13], and graphics processing units (GPUs) [14], have recently been developed to improve the computational efficiency in information science [15]. In recent years, the MIC architecture, which is a coprocessor designed for highly parallel multithreaded applications with high memory requirements, has become a widely-used HPC technology in computational biology research [16]. In this paper, we have developed a new peptide database search tool, MCtandem, that parallelizes X!Tandem based on the MIC architecture (the main accelerator of the Tianhe-2 supercomputer), via the widely adopted MPI/OpenMP protocol. MCtandem has significant advantages over previous methods that are particularly prominent when analysing large-scale datasets. The highlights are as follows:

- We design and implement an SDP-based parallel scoring algorithm using a two-level parallelization mechanism. To the best of our knowledge, MIC-SDP is the first parallel scoring algorithm for peptide identification on MIC architecture and exhibits the best execution performance.
- We adopt the MIC coprocessor for peptide database searching that uses the MIC-SDP algorithm. In design realization, we also employ asynchronous task transfer and propose a series of effective optimization strategies to decrease the communication costs between the host CPU and accelerator MIC and to balance the workload on each MIC coprocessor. The optimization strategies we use may provide insight into similar work on other database search applications.
- We also show the scalability of MCtandem by scaling the size of datasets and the number of MIC coprocessors. We obtain an ideal speedup on a multi-node cluster containing three MIC coprocessors with a total of 183 cores. The experimental results show that MCtandem has excellent scalability performance without sacrificing accuracy and correctness in the peptide database searching results.

In the following part, we first introduce the Intel MIC architecture and peptide database search method and then present the existing parallel works in peptide database searching.

***Intel MIC architecture***

Intel Many Integrated Core (MIC) architecture is a many-core coprocessor (Intel Xeon Phi coprocessor) used for highly parallel multithreaded applications that require high memory bandwidth [17]. MIC is based on an X86 Pentium core architecture but contains 512-bit-wide vector units, and each coprocessor features 61 cores clocked at 1 GHz or more, supporting 64-bit x86 instructions. The theoretical peak performance of a Xeon Phi coprocessor is up to 1 TFLOP/s in double precision. These in-order cores support four ways of hyper-threading, resulting in more than 240 logical cores [18]. In principle, one great benefit of using Intel MIC technology, compared with other accelerators and coprocessors, is the simplicity of the development. Developers do not have to learn a new programming language but may compile their source codes specifying MIC as the target architecture [19].

**Fig. 1:**
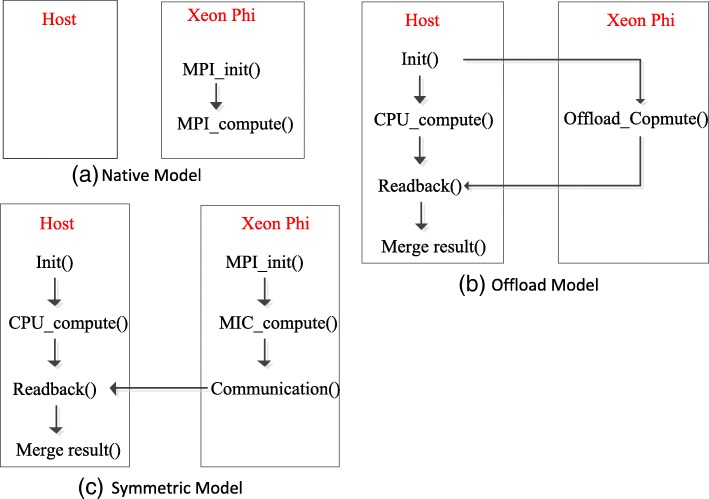
Programming models on Many Integrated Core (MIC) Architecture. **a** Native Model **b** Offload Model **c** Symmetric Model

Typically, MIC supports three kinds of programming models that can be used to design and implement parallel applications on MIC-based heterogeneous systems, as shown in Fig. 1. In its Native model, applications usually run entirely on the Intel Xeon Phi coprocessor. In its Offload model, the application starts execution on the host CPU. When an offload region is encountered, the host CPU will transfer the corresponding data to the MIC coprocessor and let the coprocessor work on it. In its Symmetric model, the host CPU and the MIC coprocessor run in parallel [18, 20]. In our work, we have used the offload model to design and implement MCtandem algorithms that can make full use of the computing resources of both the multi-core CPU and the Xeon Phi coprocessors.

***Database search-based peptide identification***

Peptide database searching is the most commonly used peptide and protein identification method and is dependent on the presence peptide sequences in a database. Essentially, all peptide sequences in the database can be scored against the experimental spectrum, and the best scoring sequence is the accepted source of the MS/MS spectrum. Sequest [2], Mascot [3] and X!Tandem [1] are some excellent algorithms in the field of peptide database searching.

The core of any protein and peptide identification method is the scoring function. In database searching, the scoring function calculates the similarity between a hypothetical spectrum and an experimental spectrum that is generated after the in-silicon digestion of protein sequences from a database, and it is the most time consuming and computationally intensive step (more than sixty percent of the total time in X!Tandem [1, 21, 22] and pFind [4]) in the flow of protein identification. Explanations are reflected in Table 1. Note that there are scoring calculations in both the model computing and model refinement steps.

**Table 1:** Time usage of X!Tandem (Second)

Time distribution	Dataset 1	Dataset 2	Dataset 3
Loading spectra	12 s	155 s	59 s
Computing models	125 s	1286 s	234 s
Models refinement	210 s	3084 s	379 s
Sorting and merging results	104 s	2574 s	298 s
Total time	451 s	7099 s	970 s
Scoring time percentage	74.3%	61.5%	63%

The scoring function is used to quantify how well a candidate peptide explains a spectrum and to choose the highest scoring peptide, which explains the spectrum the best. Among the popular protein database search approaches, the spectrum dot product (SDP) is a basic and very widely used scoring algorithm that can be used applied directly or indirectly in Sonar [23], X!Tandem [1], pFind [4] and Sequest [2], etc.

The peptide-spectrum match (PSM) is a pair (*P,S*) consisting of a peptide *P* and a spectrum *S*. The spectrum includes a list of peaks, and each peak specified by an *m*/*z* value. Therefore, representing spectra as vectors allows us to represent the generation of spectra from peptides by two-dimensional vector operations [24]. We use the boolean vector *t*=[*t*_1_,*t*_2_,…,*t*_*N*_] to represent the theoretical spectrum and *c*=[*c*_1_,*c*_2_,…,*c*_*N*_] to represent the experimental spectrum, where *t*_*i*_(*c*_*i*_) = 1 indicates that the peak *i* (*m*/*z*) (or simply the peak *i*) exists, and *t*_*i*_(*c*_*i*_)=0 otherwise. The SDP function is a kernel algorithm used to score a PSM and is defined as

1 $$ SDP = < c, t> = \sum\limits_{i=1}^{N}(c_{i}t_{i})  $$

Note that only experimental and theoretical spectra whose precursor mass distances lie within a self-defined tolerance need to be considered. We define |*E*| as the experimental spectra set and |*T*| as the theoretical spectra set. The workflow of the SDP scoring function in X!Tandem is divided into two parts, as shown in Algorithm 1. First, for each experimental spectrum (peptide), all the theoretical spectra are searched using a binary search to determine which precursor masses are within the peptide precursor mass distance and obtain*H*, assuming there are *K* spectra; Second, peak matching of the experimental spectrum and each matched theoretical spectrum (or theoretical pair) is conducted is conducted using SDP. The computation complexity is *O*(|*C*||*H*|*NK* + |*C*|*lg*(*T*)).


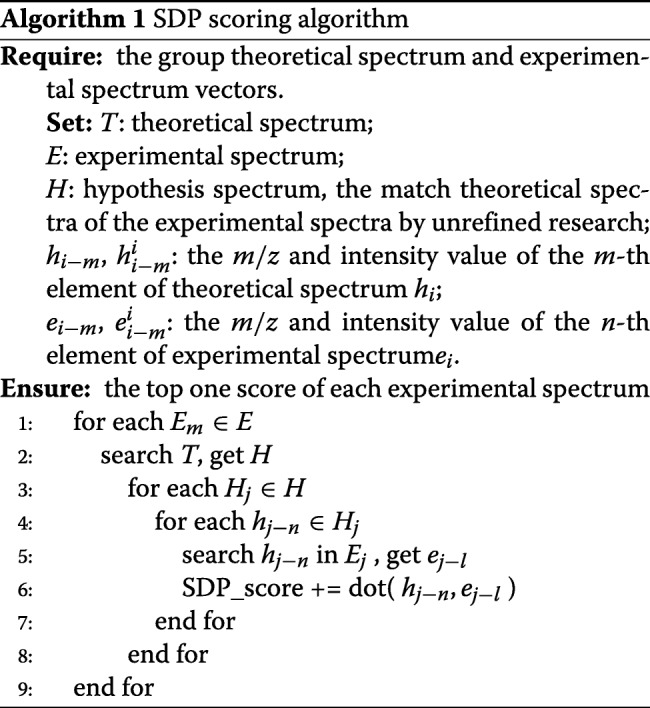


***Related research***

As one of the most powerful methods in proteomics, peptide database searching has become a focus of computational biology researchers. Recently, many efforts have been devoted to the development of efficient database search methods for protein analysis.

A notable trend is to improve the database searching scoring functions; for instance, Tang [25] adopted b/y ions and peptides and their indices to improve peptide-spectrum matching. Peng [26] and Dutta [27] used the nearest neighbour search to decrease the redundant operations in the scoring stage. Chi and Li [28] considered the problem of peptide-spectrum matching and redundant peptides and adopted an inverted index strategy to reduce the time complexity. Olivier et al.[29] developed a fast and easy-to-use tool, named X!TandemPipeline, that can process large volumes of samples simultaneously.

Using hardware acceleration is another approach to improving database search performance. Since heterogeneous computing has become a main driving force in HPC, techniques involving coprocessor acceleration have been studied for several biological data analysis methods [30]. Notably, Zhu [31] presented an efficient OpenGL-based multiple sequence alignment implementation on GPU hardware. Baumgardner [7] developed a spectrum library search algorithm based on GPU. Hussong [20] implemented a GPU-based feature detection algorithm to reduce the search time. Liu et al. [32] developed CUDA-BLASTP to accelerate BLASTP, producing identical results and maintaining the same output and input interface. Vouzis et al. [33] presented a method called GPU-BLAST, which achieves a 10-fold speedup on a GeForce GTX 295 GPU compared with the sequential NCBI-BLAST.

In addition to the GPU accelerator, using field-programmable gate array (FPGA) to accelerate the computation process is another solution with high performance. Sotiriades [34] redesigned the scoring module, which suits a single FPGA and achieves good performance. Chen Zhang [35] has built a highly efficient pipeline for coupled filtering on FPGA. In [36, 37], Dydel et al. designed a large-scale sequence analysis method on multi-FPGA platforms to explore high performance.

Additionally, some of the prevalent database search engines adopted the HPC framework [38]. X!Tandem [1] uses MapReduce [39] and MPI [21] two parallel technologies, to implement their parallel versions. Phenyx [40] and Mascot [3] adopt an MPI to build a cluster system. Among them, MR-Tandem using MapReduce achieved the best acceleration rate.

Although these methods did improve performance improvement, there are several drawbacks: the acceleration and data processing scale are still far from satisfactory for use in practical laboratories. MR-Tandem uses 50 nodes and takes 1.76 hours to complete the sequencing (Dataset: 233 MB mzXML file, including 26 172 MS/MS spectra. Database: 33 MB FASTA file, including 52 415 proteins) [39]. Large-scale heterogeneous cluster systems are based not only on common CPUs, GPUs and FPGAs, but also on different types of coprocessors. A typical representative is the more recent Intel Xeon Phi coprocessor. In this paper, we develop an improved database search tool, MCtandem, that parallelized X!Tandem to accelerate large-scale peptide identification on the CPU-MIC heterogeneous clusters.

The rest of this paper is organized as follows: “Results” section describes the experimental results by comparison with a previous study. “Discussion and Conclusion” section present our discussion and conclusions. Finally, the computational design and optimization strategies are evaluated in “Methods” section.

## Results

A series of experiments were performed to evaluate the performance and scalability of our proposed MCtandem implementation. In this section, we will first introduce the experimental environments and dataset and then compare the performance of MCtandem and some state-of-the-art peptide identification tools. Finally, we evaluate the scalability of MCtandem.

### Experimental setup

In our experiments, we implemented MCtandem using the C++ programming language and evaluated them on the MIC platform with the following configuration:

- Intel E5-2640: six-core 2.5 GHz, 15 MB SmartCache.
- Intel Xeon Phi Coprocessors 7120p: 61 hardware cores, 16 GB GDDR5 device, 1.33 GHz processor clock speed.

Tests for MCtandem were conducted using three MIC cards installed in a server with two Intel E5-2640 six-core 2.0 GHz CPU and 32 GB RAM running NeoKylin 3.2. A proper process/thread/memory affinity is the basis for optimal performance. Therefore, some default setting needs to be modified. The details of the configuration parameters are shown in Table 2. We have run X!Tandem [1] and Parallel tandem [22] on one Intel E5-2640 CPU and MR-Tandem [39] on Amazon Web Services.

**Table 2:** A representative job script

Script commands
module load craype-hunepages2M
export MKL_FAST_MEMORY_LIMIT = 0
export OMP_PROC_BIND = TRUE
export OMP_PLACES = threads
export OMP_STACKSIZE = 512m
export OMP_NUM_THREADS = 16

We scanned two protein sequence databases: the 5.2GB UniProtKB/SwissProt (540 171 proteins) and the 18GB UniProtKB/TrEMBL (1 821 879 proteins). The protein sequence database is obtained from the UniProtsKB database (http://www.UniProt.org/downloads/), which is a non-redundant, high quality, and manually annotated protein sequence database [41]. The experimental spectra data were generated by tandem spectrometry experiments that analysed the behaviour of a mixture with human liver. More details are shown in Table 3.

**Table 3:** Test datasets for MCtandem

Dataset	Instrument	Enzyme	Tolerance	Modifications	Size
Dataset 1	LTQ	Trypsin	Precursors: 3Da Fragment: 0.5Da	Fixed: Cabamidomethylation (C)	51.5MB (18 172 spectra)
Dataset 2	QSTAR	AspN	Precursors: 2Da Fragment: 0.2Da	Fixed: Cabamidomethylation (C)	272MB (52 503 spectra)
Dataset 3	LTQ	LysC	Precursors: 0.2Da Fragment: 0.5Da	Fixed: Cabamidomethylation (C)	486MB (106 616 spectra)

### Performance on a single MIC node

First, we compared the single-MIC performance of the proposed MCtandem implementation to that of X!Tandem. For single MIC card tests, we used the UniProtKB/Swiss-prot a test database and measured the total search time to calculate the computing speedup values. To enhance the accuracy of the results, three different datasets (see Table 3) were used in the experiments.

**Fig. 2:**
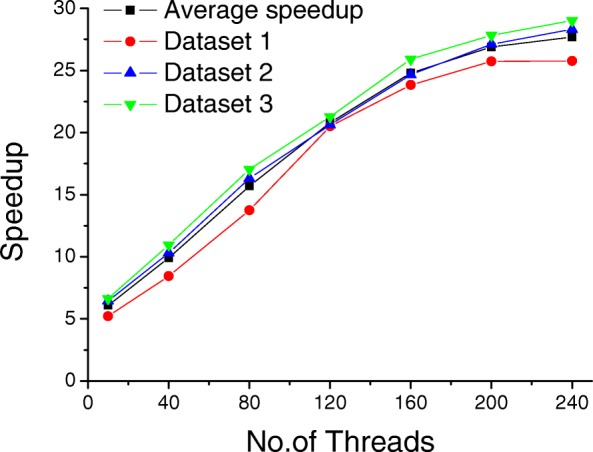
Speedup effect of SDP using a single MIC coprocessor

Table 4 shows the corresponding computing time and speedup of MCtandem and X!Tandem. MCtandem is executed on a single MIC node. X!Tandem is executed on an Intel E5-2640 CPU with 32 threads. This table shows that Dataset 1 achieved a 25.77-fold speedup, Dataset 2 achieved a 28.31-fold speedup and Dataset 3 achieved a 29.02 timeless speedup. The speedup is achieved from the parallel MIC-SDP scoring algorithm and optimization techniques. In addition, we have also tested the impact of thread count on the speedup of MCtandem by changing the amount of threads. The experimental results show that it can run up to 29 times faster on a single MIC than the original CPU-based version, as shown in Fig. 2.

**Table 4:** Speedup effect of SDP using a single MIC coprocessor

Software	Dataset 1	Dataset 2	Dataset 3
X!Tandem	451 s	7099 s	970 s
MICtandem	17.5 s	251 s	34 s
Speedup	25.77	28.31	29.02

**Fig. 3:**
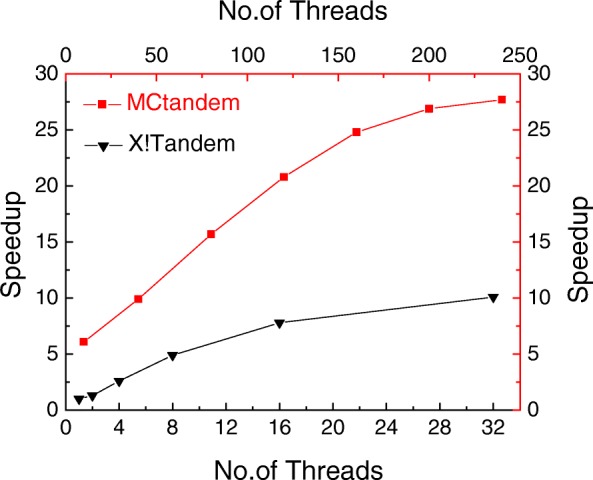
Comparisons of performance between MCtandem and X!Tandem. Comparisons of performance between MCtandem and X!Tandem: when the number of the threads reaches the 240, MCtandem can speed up about 28 times over

We further compare the obtained speedup of the Parallel tandem [22] on the multi-core CPU and MCtandem on a single MIC. Parallel tandem is a parallel version of X!Tandem using PVM. For testing Parallel tandem on the multi-core CPU, we limited the number of threads to twice of the core, two CPUs every 8 cores. The MIC architecture’s in-order cores support four-way hyper-threading, with more than 240 logical cores. Figure 3 reports the speedup of MCtandem and Parallel tandem against the number of threads. From this figure, it can be observed that MCtandem can achieve nearly 28-fold speedup over the X!Tandem, while Parallel tandem running on multi-core CPU can obtain nearly a 9-fold speedup.

### Performance on the MIC cluster

**Fig. 4:**
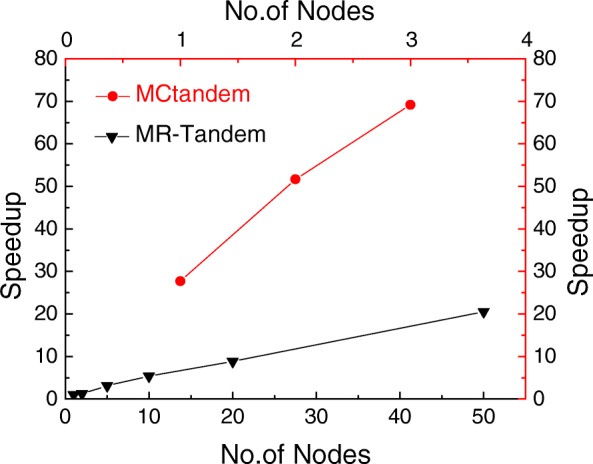
Comparisons of performance between MCtandem and MR-Tandem. MCtandem takes only 3 nodes to achieve 61.7-fold speedup

To evaluate the performance of multi-node acceleration, we used three nodes as the test platform. Each node is equipped with two 6-core Intel E5-2640 CPUs and a 61-core Intel Xeon Phi coprocessor. Figure 4 gives the speedup of MCtandem compared with MR-Tandem, where the X axis represents the number of nodes in the MIC cluster and the Y axis represents speedup. MR-Tandem uses 50 nodes to obtain a 20.56-fold speedup, while MCtandem takes only 3 nodes to achieve 61.7-fold speedup. MCtandem shows significantly better performance than MR-Tandem as the number of nodes increases. The results indicate that MCtandem exhibits good scalability in terms of the number of computing nodes.

### Performance for processing large-scale datasets

**Fig. 5:**
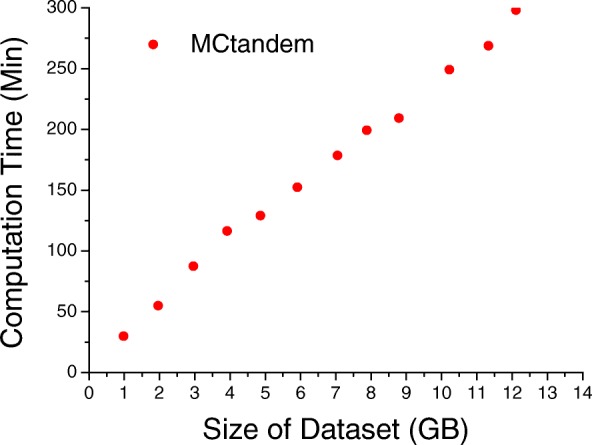
Performance of MCtandem on datasets sized 0.98-12.11GB

In the large-scale experiments, we tested the capacity of big data processing by varying the size of the dataset. The large datasets in the experiment were formed by merging Dataset 1, Dataset 2, and Dataset 3. X!Tandem and MR-Tandem cannot operate normally for datasets larger than 1.96 GB in 18 GB databases. We ran MCtandem on a single MIC node. Figure 5 demonstrates the performance of MCtandem as the dataset size increases from 0.98 GB (210 252) to 12.11 GB (3 102 956 spectra). As shown in Fig. 5, MCtandem can handle extremely large datasets with a linear increase in computation time with dataset size. For a 12.11 GB dataset, MCtandem took 282 min, which is acceptable in most practical laboratories. Our implementation also demonstrates good scaling in terms of dataset size.

## Discussion

To overcome the drawbacks of the existing protein database search methods, we propose a new algorithm MCtandem, which parallelizes X!Tandem based on the MIC cluster via the widely adopted MPI/OpenMP protocol. The MCtandem has significant advantages over the previous methods that particularly show when analyzing large-scale spectra datasets. In this section, we first validate our results with a previous study and then evaluate the performance of optimization technology used in MCtandem.

### Accuracy analysis

We verified the accuracy of MCtandem by comparing the cosine values for MCtandem to those of X!Tandem. The results are presented in Table 5. MCtandem and MR-Tandem obtain the same cosine value for the spectra datasets. This result proves that the searching results obtained by MCtandem are consistent with those obtained by MR-Tandem. This result validates that MCtandem achieves much higher execution performance than MR-Tandem without sacrificing the accuracy and correctness of the results.

**Table 5:** Accuracy analysis of MCtandem

Dataset	Cosine of MR-Tandem	Cosine of MCtandem
Dataset 1	0.975684	0.975684
Dataset 2	0.992485	0.992485
Dataset 3	0.982365	0.982365

### Summary of optimization technology

To test the effectiveness of the above optimization, we ran MCtandem on MIC cluster for three nodes and searched Dataset 2 (the data size is 486 MB, including 106 616 spectra) in the UniProtKB/TrEMBL database. The optimized MCtandem gained a 23.4 percent performance boost compared with the original MCtandem, as shown in Table 6. We also tuned the communication across nodes on MIC clusters to achieve the best utilization of various computing power within the heterogeneous system. Meanwhile, the optimization methods we use may provide insights into other database search applications.

**Table 6:** Computational Time Before and After Optimization

Methods	Execution time (seconds)	Benefits
Before Optimization	1553 s	0
Pre-Fetching	1391 s	10.1%
Multithreading and Hyper-Threading	1408 s	9.3%
Vectorization	1329 s	14.4%
With Both Optimization	1190 s	23.4%

## Conclusion

As the amount of MS/MS data increases rapidly, the prohibitive computing time required for large-scale peptide identification has become a critical concern in proteomics. In this paper, we design and implement a parallel scoring algorithm to accelerate large-scale peptide identification on CPU-MIC heterogeneous clusters. To achieve high performance, we reformulated the scoring model to reduce the time complexity and eliminated the data dependence to enable all possible localities and vectorization. Performance is also tuned among the CPU and Xeon Phi coprocessors by pre-fetching, multithreading and hyper-threading, vectorization and communication overlapping schemes to achieve the optimum performance and best utilization of various computation resources within heterogeneous systems. Evaluations on real MS/MS spectra datasets show that MCtandem achieved a 28-fold speedup on a single MIC. Our experimental results also demonstrate that MCtandem can significantly increase the performance and scalability of large-scale peptide identification without sacrificing correctness and accuracy in the result. We believe that the techniques we use may provide insights into similar work on other large-scale sequence analysis applications.

## Methods

### Computational Design

We first analysed X!Tandem to chase down the hotspot of the program and then profiled the performance of X!Tandem by using Intel VTune TMAmplifier XE. The result shows that the “mscore” function (the calculation of the sequence similarity scores) represents more than 60 percent of the whole computation time and should therefore be accelerated to improve performance. Meanwhile, we found that when searching the same type of MS/MS spectra, X!Tandem processes each experimental spectrum individually, which is desirable for parallel processing.

Based on these findings, our MCtandem on the MIC heterogeneous system requires a two-level parallelization mechanism to implement multi-level parallelism, which specifically includes: task-level parallelism between CPUs and their MIC coprocessors using a dynamic task scheduling method and thread-level parallelism employing sequence-decomposition through dynamically scheduled multithreading.

#### Parallelization between CPU and MIC

In the Offload model, the task assignment between the host CPU and the MIC coprocessor should be considered. Since the MIC coprocessor has a disjoint memory space from the host CPU, task allocation would incur data transfer. To support search tasks for large-scale peptide databases, we further divide each spectra subset into a set of chunks. We design and implement a task-level dynamic distribution framework to distribute these chunks to both the host CPU and the MIC coprocessors.

**Fig. 6:**
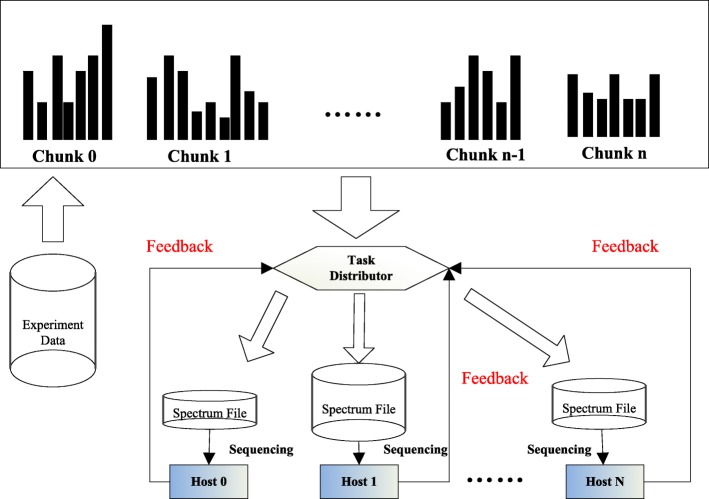
Framework of dynamic task distribution

As shown in Fig. 6, first, a sample test is executed to explore the computational source of all computing nodes. Then, based on information about the sample data run time and load balancing, the performance factors of different computing nodes are automatically collected. The relevant details are described in the next paragraph. Finally, with the performance factor of each node, we can then calculate and adjust the appropriate size of the spectra chunk assigned to the corresponding node using a dynamic feedback task scheduling algorithm [42].

To balance the load dynamically and eliminate the system bottleneck, we must choose appropriate load parameters for the performance factor. The first aspect to consider is CPU utilization. In our implementation, we extracted the real-time information parameters in a /proc/stat file of the Linux system to calculate CPU utilization. The task queue length of a single core decide whether the task scheduler can keep up with the system requirements, if it is too long, the execution time of a job will become too long, which causes the system to be in the state of overload. Therefore, the average length of the task queue is another key performance factor. We can use related parameters in file /proc/loadavg of the Linux system to reflect the average task queue length of a single core. In heterogeneous systems, memory utilization needs to be monitored. Four useful items are extracted from the file /proc/meminfo: free memory (MF), file cache (Cached), total memory size (MT), and block-device buffers (Buffers). Memory utilization is defined as MemUsage, which can be calculated by

2 $$ \begin{aligned} MemUsage = \frac{MT - MF - Buffers - Cache}{MT}\\ \end{aligned}  $$

The dynamic feedback task scheduling process is described as follows:

Step 1 Users choose a host node in the computing environment service as the task scheduling host node.
Step 2 The scheduling host node uses configuration requirements to filter static resource information and access the real-time information of backup computing resources through the network.
Step 3 The scheduling host node distributes tasks to the computing node, monitors the execution status of the search task and collect the computing results.
Step 4 According to the ratio of the number of remaining hosts after overload exceeded the number of backup hosts, the geometric weighted coefficient was adjusted, returning to Step 2. The task is complete when the load on each node is balanced.

Our experimental results show that dynamic task scheduling can maintain the system load imbalance below 8 percent in most cases.

#### Parallelization across MIC coprocessors

Due to the high bus bandwidth between CPU and system memory, CPU can process data input and output very quickly. Unlike the CPU, MIC coprocessor threads can process multiple database peptide batches in parallel. However, because of the relatively low bus bandwidth between the system memory and the MIC coprocessor, data read back from the MIC coprocessor to the CPU is a known bottleneck and should be minimized. In this work, we design a hybrid scoring algorithm and employ the peptide sequence-decomposition method to implement thread-level parallelism.

Each core in MIC is an dual-issue, in-order core, which has four-way hyper-threading supports to improve multi-cycle instruction latency and hide memory. Our MIC-SDP scoring algorithm on MIC is designed so that each MIC core deals with one experimental spectrum serially, scoring with its entire matched theoretical spectrum. Compared with the original SDP algorithm, the improvements in MIC-SDP are as follows: First, the MIC-SDP scoring algorithm dispensed with the first loop in the SDP algorithm by allocating each experimental spectrum to a thread, which significantly decreases the compute time as many threads are about working in parallel. Second, the MIC-SDP algorithm merges the SDP calculation and the peak matching steps to decrease the space for the variable. As shown in Algorithm 2, the computational complexity of MIC-SDP decreases to *O*(*lg*(*T*)+|*H*|*NK*).


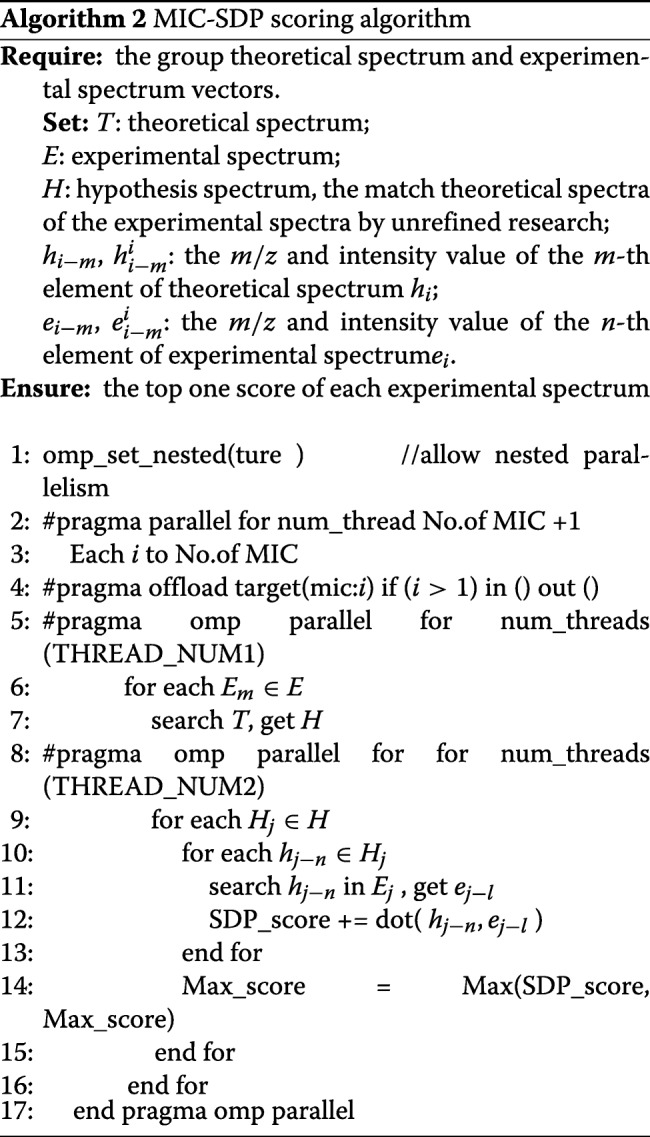


When the computation tasks (scoring module) are offloaded to the MIC coprocessor, it spawns a set number of threads to accomplish these tasks (depending to the number of MIC cores). These threads develop the parallelism of the scoring tasks through the peptide sequence-based decomposition method, where each thread acquires works based on the peptide sequence units. Meanwhile, these threads adopt a dynamical scheduling policy for workload balancing, where each thread acquires a new sequence from the unsettled peptide sequence pool after processing every peptide sequence.

Our MCtandem algorithm caters to the MIC architecture in deploying SDP-based scoring with MPI+OpenMP. It can fully utilize the vector processing unit (VPU) hyper-threading. Meanwhile, to maximize MCtandem’s overall processing capacity and achieve loading balance in the MIC cluster, we employed dynamic task scheduling to automatically move spectra data from overutilized to underutilized VPUs.

**Fig. 7:**
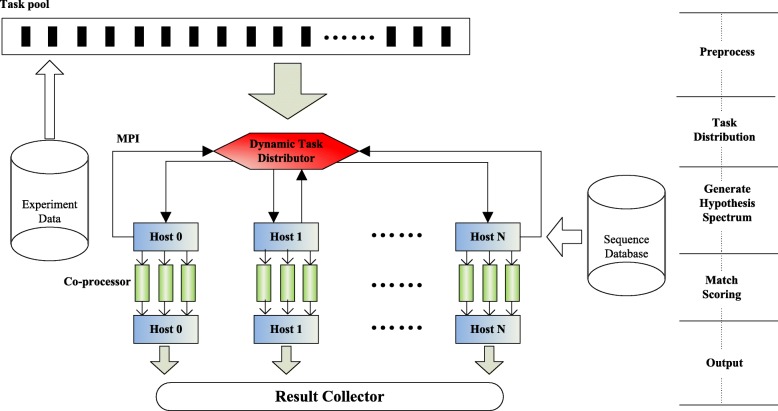
The overall flow of MCtandem. Our two-level parallelization scheme on the CPU-MIC heterogeneous system combines: (1) task-level parallelism between CPU and MIC using a dynamic task scheduling method (based on MPI). (2) thread-level parallelism employing sequence-decomposition through dynamically scheduled misreading (based on OpenMP)

The workflow description of MCtandem is presented in Fig. 7. To fully exploit the heterogeneous system on the MIC, we defined four phases in the execution of MCtandem. In the first phase, MCtandem partitions an MS/MS spectra dataset into appropriately-sized datasets and distributes them across multiple computing nodes based on MPI scheduling. In the second phase, the hypothesized spectra dataset is obtained through an unrefined search on Xeon E5 CPU. In the third phase, MCtandem distributes each mass spectrum and the corresponding hypothetical spectra dataset to the Xeon Phi coprocessor. Each VPU addresses one experimental spectrum using our MIC-SDP algorithm. In the last phase, the output files are combined into a results document.

### Optimization techniques

Several optimization techniques are employed on MCtandem, including pre-fetching, multithreading and hyper-threading, vectorization, computation and communication overlapping schemes.

#### Pre-fetching

Task assignment incurs data transfer and memory access, which greatly reduces the parallel efficiency, because the MIC coprocessor has a disjoint memory space from the host CPU. We implemented the pre-fetch manually by using a tightly-coupled methodology to divided tasks between the CPU and MIC and further improved the parallel efficiency. We implemented the double-buffering mechanism, which is a technique designed to improve performance by hiding memory access, as shown in Algorithm 3. When there are multi-cycle DMA read (write) operations, MIC coprocessors assign double the memory space in the scratch pad memory to two sets of spectra. The two spectra are buffered from each other. When one spectra is scoring, the other spectrum serves as the message buffer.


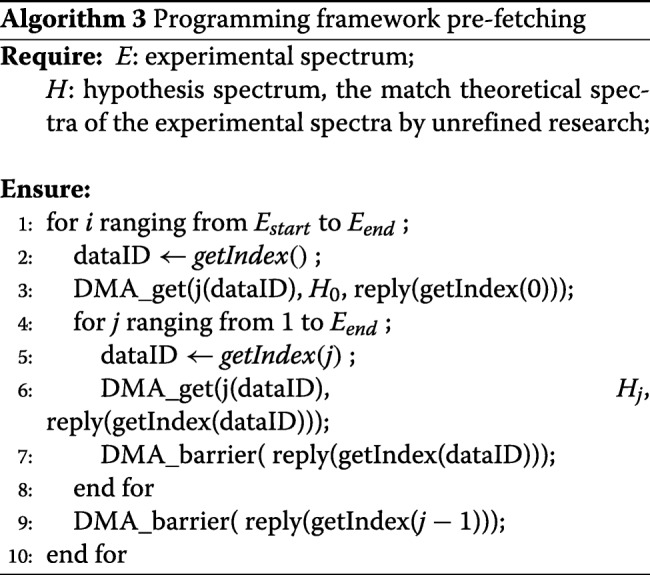


#### Multithreading and hyper-threading

Running code outside the parallel scaling region either slows down scientific productivity or wastes valuable computing resources. An appropriate parallel/thread scaling of applications is critical to run the codes efficiently in HPC systems. We found experimentally that the MCtandem performs best with four or eight threads per MPI task at all node counts for all datasets. For the runs with small node clusters (one or two nodes), using four threads per MPI task performs best. However, when the node clusters increase, using eight threads per MPI task outperforms four threads per MPI task. Consequently, we recommend using eight threads per MPI task or more for larger threads.

Hyper-threading could improve the application acceleration performance through increasing resource utilization by simultaneously running multiple threads/processes on the hardware threads on the core, making effective use of the cycles that would otherwise be wasted due to branch mis-predictions, data dependencies, cache misses, and/or waiting for other resources in a single thread/process execution on the core [43]. With the MIC, which provides four hardware threads per core, hyper-threading improved MCtandem’s performance slightly.

#### Vectorization

In heterogeneous MIC architecture, the host CPU and the MIC coprocessor share a similar computing architecture that consists of VPUs and multiple cores. Therefore, vectorization is a key point in the optimization process. In this work, we have achieved efficient utilization of all available computing resources by utilizing vectorization. To implement vectorization optimization means that several spectra can be processed together. In database searching, the hotspot of database searching is the calculation of match scoring of pairwise spectra, which can be vectorized by the same methods, including numerical calculation and copying, demanding numeric calculation, and vectoring the copy to execute in parallel. Meanwhile, in accordance with vectorization, we modified all dependent statements to ensure a better vectorization. As the key to the entire optimization process, the vectorization technique achieved a performance of 67.21 Gflops.

#### Communication overlapping scheme

With the efficient MPI/OpenMP parallelization of large-scale peptide database searching, our MCtandem algorithm based on a heterogeneous system not only makes efficient use of the host CPUs and MIC coprocessor resources as described previously but also exploits communication overlapping to minimize the communication latency. During the MCtandem’s implementation, we adopted an improved proxy infrastructure [44] to promote the communication performance and scalability of MCtandem on MIC Clusters.

**Fig. 8:**
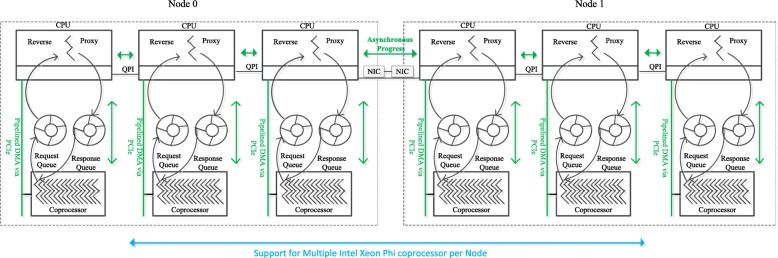
The improved proxy infrastructure. Our proxy infrastructure can be extended to support a single MPI process per Intel Xeon Phi for external communication, and OpenMP threads for parallelism within the Intel Xeon Phi coprocessor

Two memory-mapped circular queues between the host and the MIC coprocessor used in our design are shown in Fig. 8. One queue is used for requests and one for responses. To eliminate the need to use lock and unlock operations between the host CPUs and the MIC coprocessor, a set of free slots to hold the communication commands, and each queue maintains head and tail pointers to determine whether the queue is full or empty. More specifically, before putting a task on MIC coprocessor, the proxy obtains a free slot by comparing the trailer and header pointers to ascertain if the request queue is full. The queue will wait for an available slot if it is full. In the meantime, this slot will then increment the tail pointer and the corresponding will fill with the intended command. Moreover, to ensure the spectra data pipes between the local CPU and the local MIC coprocessor, between the remote CPU and the local CPU, and between the remote MIC coprocessor and the remote CPU am always busy receiving or sending data, the sending and receiving of spectra data chunks is performed in a pipelined manner.

## Data Availability

The software, the test dataset and the parameter settings are available from https://github.com/LogicZY/MCtandem. More experimental spectra data can be obtained from the Iprox (https://www.iprox.org/). The protein sequence database is obtained from the UniProtsKB database (http://www.UniProt.org/downloads/).

## Abbreviations

DMA: Direct memory access
FPGA: Field programmable gate array
GPUs: Graphics processing units
HPC: High performance computing
MIC: Many integrated core
MPI: Message passing interface
MS/MS: Tandem mass spectrometry
PSM: Peptide-spectrum match
PTMs: Post-translational modifications
SDP: Spectrum dot product
VPU: Vector processing unit
